# Integrated investigation and discovery of therapeutic targets for 3-hydroxybakuchiol against diabetes based on molecular docking studies and cell experiments

**DOI:** 10.1186/s12906-023-04248-6

**Published:** 2023-11-29

**Authors:** Min Liu, Xinyu Wang, Junsong Yang, Dan Qin

**Affiliations:** School of Basic Courses, Bengbu Medical College, Bengbu, 233000 China

**Keywords:** HepG2 cells, 3-Hydroxybakuchiol, Molecular docking, Glucose metabolism

## Abstract

**Background:**

Diabetes mellitus is a prevalent endocrine condition. We aimed to investigate the anti-diabetic effects of 3-hydroxybakuchiol (HYD) by exploring its potential targets and molecular mechanisms through bioinformatics analysis and cell experiments.

**Methods:**

We performed an extensive search and screening of HYD and its potential targets for diabetes mellitus across various databases. Enrichment analyses were conducted using the ClusterProfiler package. PPI networks of the identified genes were constructed using STRING, and topological analysis was performed to identify core targets. The results were further confirmed through molecular docking. To validate the findings of our bioinformatics analysis, we conducted cell experiments using insulin resistance-induced HepG2 cells and C2C12 cells.

**Results:**

We discovered 260 common targets of HYD and diabetes mellitus, which were primarily related to the MAPK signaling pathway, PI3K-Akt signaling pathway, and endocrine resistance. A topological analysis of the PPI network identified four core targets (HSP90AA1, AKT1, SRC, and MAPK1). Molecular docking studies further confirmed the strong binding ability between HYD and these core targets. In cell experiments, we observed that HYD enhanced glucose uptake and suppressed gluconeogenesis in HepG2 cells and C2C12 cells. This resulted in an improvement in glucose metabolism, potentially through the regulation of the PI3K-Akt pathway.

**Conclusions:**

This study provides valuable insights into the pharmacological effects of HYD on diabetes mellitus, suggesting its potential as a promising treatment option for the disease.

**Supplementary Information:**

The online version contains supplementary material available at 10.1186/s12906-023-04248-6.

## Introduction

Type 2 diabetes mellitus (T2DM) is a complex metabolic disease linked to beta-cell dysfunction, insulin resistance, and impaired insulin secretion [1]. This results in persistent hyperglycemia and can lead to serious health complications such as cardiovascular diseases, nephropathy, retinopathy, neuropathy, and cognitive impairment [2–4]. The global prevalence of T2DM was 435 million in 2019 and is projected to increase to 578 million by 2030 and 700 million by 2045 [5]. Despite research efforts, effectively managing T2DM and its complications remains challenging [6, 7]. Current strategies prioritize regulating blood glucose levels using synthetic insulin and hypoglycemic drugs, but these can have harmful effects and drug resistance [8]. Therefore, there is increasing interest in identifying low-cost natural products with minimal side effects for treating metabolic disorders, including T2DM [9, 10].

Bakuchiol, a prenylated monoterpene extracted from Psoralea corylifolia seeds, has various pharmacological effects, including hypoglycemic, anti-depression, anti-cancer, anti-inflammatory, and antibacterial effects [11–15]. It is effective in treating diabetic cardiomyopathy caused by hyperglycemia [16]. In addition, a previous study has confirmed that orally administering bakuchiol reduces blood glucose levels in db/db mice with type 2 diabetes [15]. A compound called 3-hydroxybakuchiol (HYD) with a similar structure to bakuchiol has been discovered in Otholobium mexicanum, showing potential anti-diabetic activity [17, 18]. This suggested a potential anti-diabetic activity of HYD. However, there have been no systematic studies on the anti-diabetic activity of HYD.

The use of network pharmacology has gained popularity in drug research due to advancements in bioinformatics and related databases. This approach allows researchers to uncover relationships between drugs, pathways, and targets [19, 20]. Molecular docking analysis can validate the potential links between active components and target genes identified through network pharmacology analysis [21]. This approach provides a comprehensive understanding of the interactions between active components and genes associated with diseases, aiding in the development of potential therapeutic interventions.

**Fig. 1:**
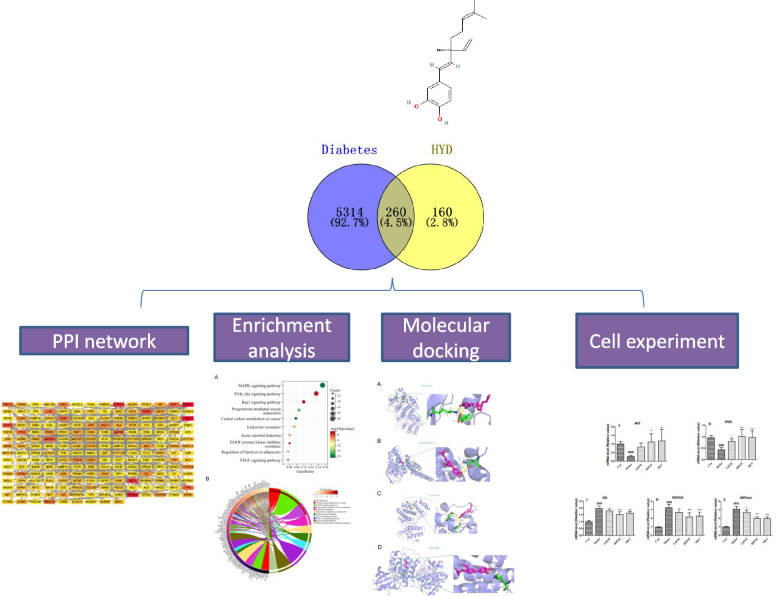
Workflow of the present study

In this study, we employed a network pharmacology approach, along with molecular docking technology and cell experiments, to investigate the biological pathways linked to the therapeutic effects of HYD in managing diabetes. This study is the first to thoroughly explore the mechanisms and effects of HYD, leading to a solid theoretical basis for its clinical use. Figure 1 illustrates the workflow process in a schematic chart.

## Materials and methods

### Collection of HYD-related targets

All the targets of HYD have been obtained by using the following three databases: Super-PRED (https://prediction.charite.de/index.php), PharmMapper (http://www.lilab-ecust.cn/pharmmapper/), and SwissTargetPrediction (http://www.swisstargetprediction.ch/). Related genes were obtained after the duplicated genes were removed.

### Collection of diabetes-related targets

To identify targets related to diabetes, we performed a comprehensive search using the keyword “type 2 diabetes” on three databases: GeneCards (https://www.genecards.org/), Therapeutic Target Database (https://db.idrblab.net/ttd/), and Comparative Toxicogenomics Database (https://ctdbase.org/). Duplicate targets were removed and a list of targets associated with diabetes disease was obtained.

Construction of protein-protein interaction (PPI) network and identification of core targets.

To identify target genes related to both HYD and diabetes, we utilized the Venn tool for analysis. This allowed us to determine the target genes of HYD that are also associated with diabetes. Subsequently, we utilized the STRING database (https://version-12-0.string-db.org/) to construct a PPI network for these shared targets. The PPI network was then imported into Cytoscape (version 3.7.2) software for visual analysis, and the cytoHubba plug-in was employed to screen for critical targets within the network [22].

### Functional enrichment analysis

To further investigate the mechanism of HYD in treating diabetes, we conducted GO function and KEGG signaling pathway enrichment analysis in this study [23, 24]. The ClusterProfiler package (version 2.2.7) was obtained from Bioconductor, and R software was utilized to perform GO and KEGG enrichment analysis on the shared targets, with a significance threshold set at *p* < 0.05.

### Molecular docking analysis

In this study, the interactions between HYD and the hub genes were investigated using the molecular docking approach. The 3D structure of the target protein was obtained from the PDB database. PyMOL (version 2.5.5) was used to eliminate the water molecules and original ligands of the target protein. We utilized ChemDraw 19.0 software to draw the molecular structures of small molecule compounds individually for ligand molecule processing. The resulting format files were then imported into Chem3D 19.0 software to optimize the energy using the program, and saved in PDB format. Next, we imported the PDB format file into AutoDockTools 1.5.6 software to adjust the ligand charge and saved it as a PDBQT file. Subsequently, AutoDock software (1.5.6) was employed for molecular docking analysis. Here is a detailed procedure for molecular docking: the AutoDockTools 1.5.6 software was used to open the PDBQT file of the receptor macromolecule and ligand. To identify the active binding site for molecular docking, we utilized the coordinates of the co-crystallized ligand. After identifying the active pocket of the target protein, we proceeded to define the coordinates and size of the grid box. The parameters for the grid box were as follows: a spacing of 0.5 Å, 80 points in the x-dimension, 80 points in the y-dimension, and 80 points in the z-dimension. The docking process utilized the Vina force field and the Lamarckian GA (4.2) algorithm. Throughout this study, 100 GA runs were performed while maintaining the default settings for other docking criteria. The results were then visualized using PyMOL. Furthermore, information regarding the absorption, distribution, metabolism, excretion, and toxicity (ADMET) characteristics of HYD was acquired from the pkCSM database, accessible at http://biosig.unimelb.edu.au/.

### Cell culture

HepG2 cells and C2C12 myoblasts (Chinese Academy of Medical Sciences, Beijing, China) were cultured in Dulbecco’s modified Eagle’s medium supplemented with 10% FBS and 100 IU/mL penicillin-streptomycin. The cells were incubated at 37 °C in a 5% CO2 environment. To induce the insulin resistance model, we followed the protocol outlined in the previous study [25]. HepG2 or C2C12 cell lines were seeded in 96-well plates and cultured for 24 h. Following this, they were exposed to a 12-hour serum-free medium for starvation treatment. The cells were subsequently washed three times with PBS, and then treated with Dulbecco’s modified Eagle’s medium supplemented with 100 nM insulin and 25 mmol/L glucose at 37 °C in a 5% CO2 atmosphere for 24 h.

### Drug treatment

The study involved dividing cells into five groups: control, model, HYD low dose (LHYD, 25 µg/mL), HYD high dose (HHYD, 50 µg/mL), and metformin (MET). Cells in the control group were maintained in Dulbecco’s modified Eagle’s medium. To induce insulin resistance, cells in the model, HYD, and MET groups were subjected to the method mentioned above. After induction, the HYD groups were treated with either a low or high dose of HYD, while the MET group was treated with a dose of 10^−3^ mol/L. All cells were then incubated at 37 °C in a 5% CO_2_ atmosphere for 24 h.

### Cell viability

The viability of cells treated differently (HYD) for 24 h was assessed using the CCK-8 test kit (Shizebio, Shanghai, China) based on the instructions of the manufacturer.

### Glucose consumption assay

Glucose consumption was evaluated using a modified version of the methodology described in a previous study [26]. HepG2 cells or C2C12 cells were induced to develop insulin resistance and then treated with different concentrations of HYD or MET as previously mentioned. Glucose assay kits (glucose oxidase method) were used to quantify glucose consumption in the medium (Nanjing Jiancheng Bioengineering Institute, China).

### Quantitative real-time polymerase chain reaction (qRT-PCR)

To perform qRT-PCR, total RNA was extracted from cells using TRIzol Reagent (Thermo Fisher Scientific, USA) following the manufacturer’s protocol. The extracted RNA was then converted into cDNA using cDNA synthesis kits (Thermo Fisher Scientific, USA). We conducted the qRT-PCR analysis on a StepOnePlus Real-time PCR system (Applied Biosystems, CA, USA) and determined the mRNA expression levels using a 2^–ΔΔCt^ method. The primer sequences used for qRT-PCR can be found in Table S1.

### Statistical analysis

Data analysis was performed using GraphPad Prism 8.0 software. Experimental results are presented as mean ± standard deviation (SD). Group differences were compared by one-way analysis of variance. Statistical significance was defined as *P* < 0.05.

## Results

### Identification of potential targets of HYD treating diabetes

**Fig. 2:**
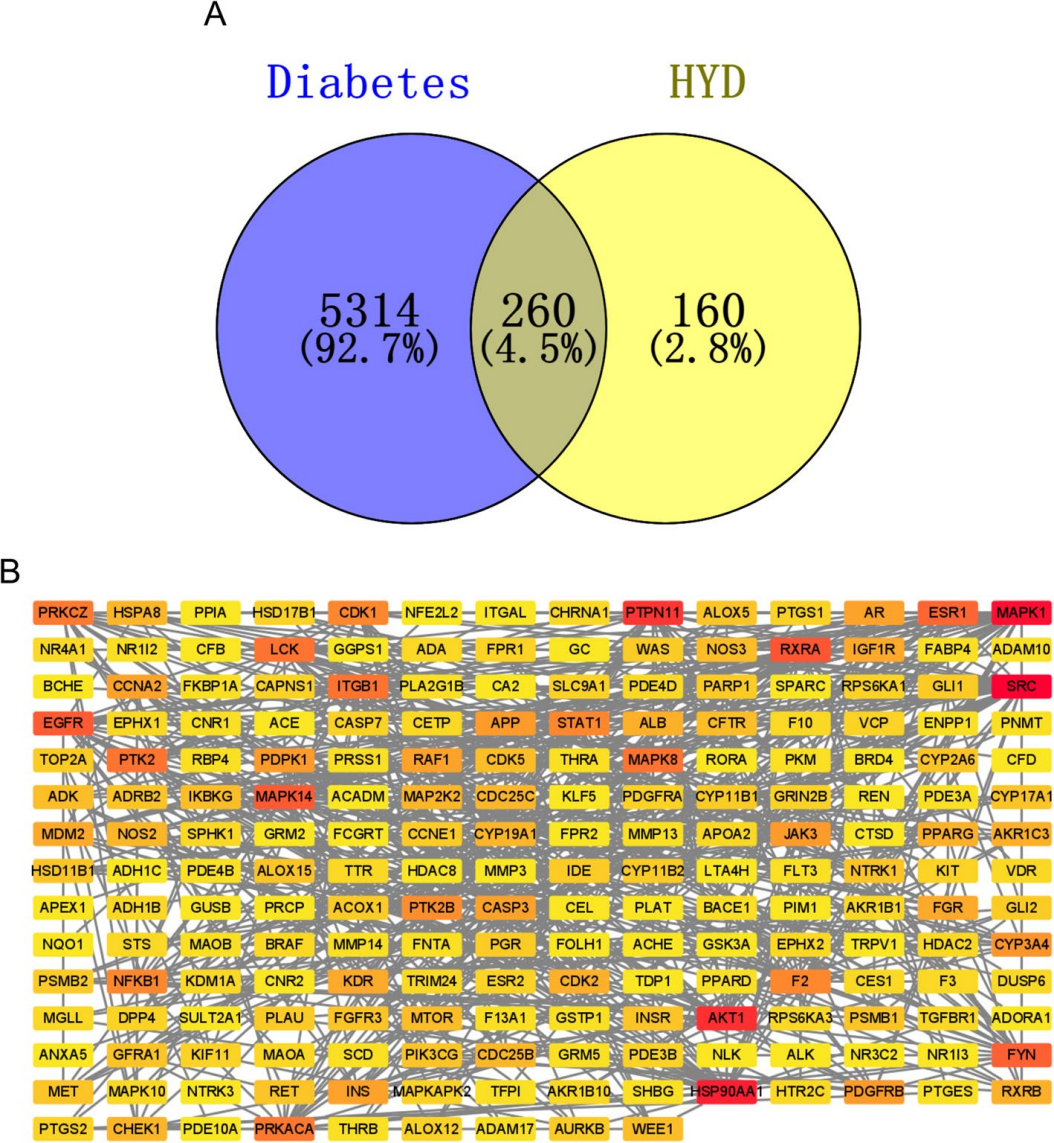
Identification of potential targets of HYD treating diabetes. **A** The Venn diagram of diabetes and HYD. **B** The protein-protein interaction (PPI) network of potential targets of HYD for diabetes was constructed using the Cytoscape software. Each node in the network represents a specific target, while the edges represent the protein-protein associations, including known interactions. Nodes that are colored red indicate a higher degree, while those colored yellow represent a lower degree

As presented in Fig. 2A, we collected 5574 diabetes-related targets from GeneCards, the Therapeutic Target Database, and the Comparative Toxicogenomics Database. A total of 420 HYD-related targets were obtained from Super-PRED, PharmMapper, and SwissTargetPrediction databases. A total of 260 targets were identified as potential targets of HYD in the treatment of diabetes. To further investigate the interactions between these targets, a PPI network was constructed using Cytoscape software. The resulting PPI co-expression network had 205 nodes and 590 edges, as shown in Fig. 2B.

### Functional enrichment analysis

**Fig. 3:**
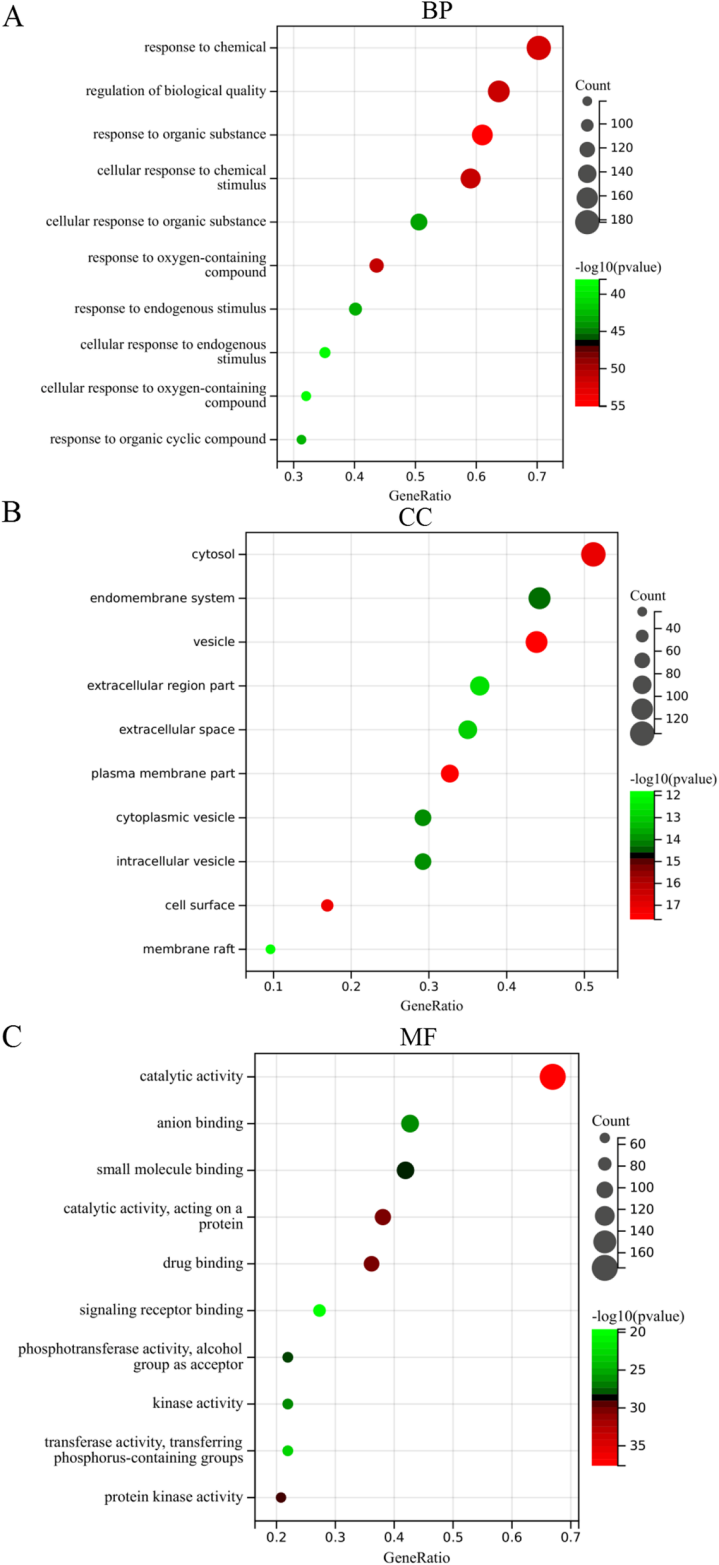
The GO enrichment analysis of HYD in the treatment of diabetes

**Fig. 4:**
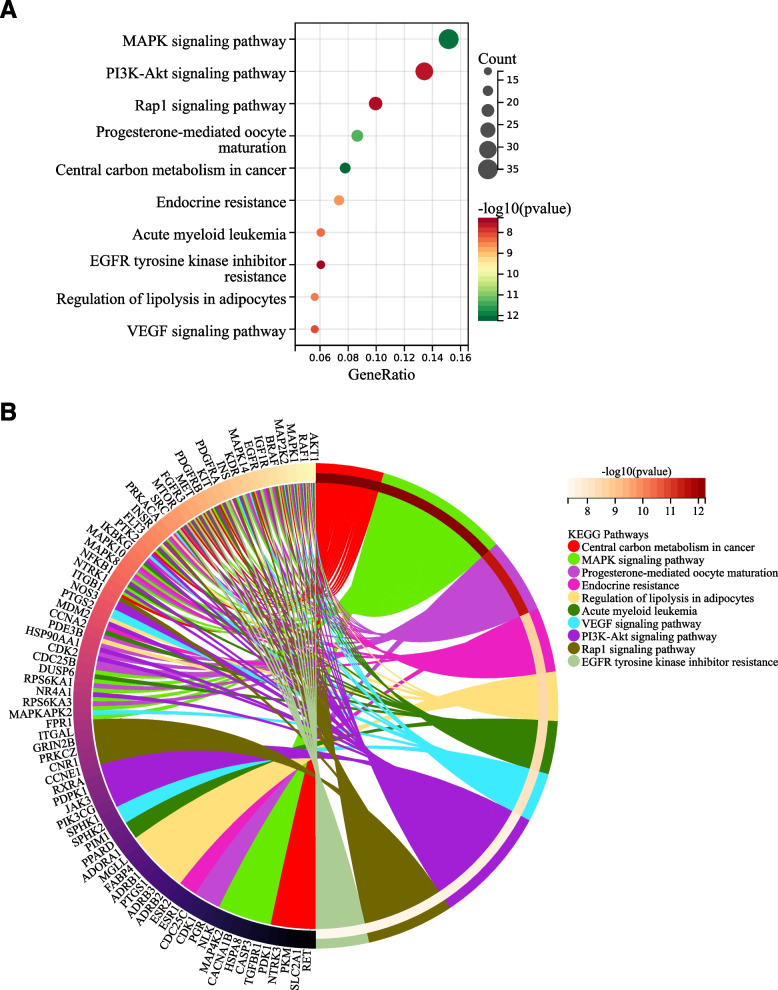
The results of KEGG enrichment analysis of HYD in the treatment of diabetes. Bubble (**A**) and circle (**B**) diagrams showing the top 10 enriched pathways

**Fig. 5:**
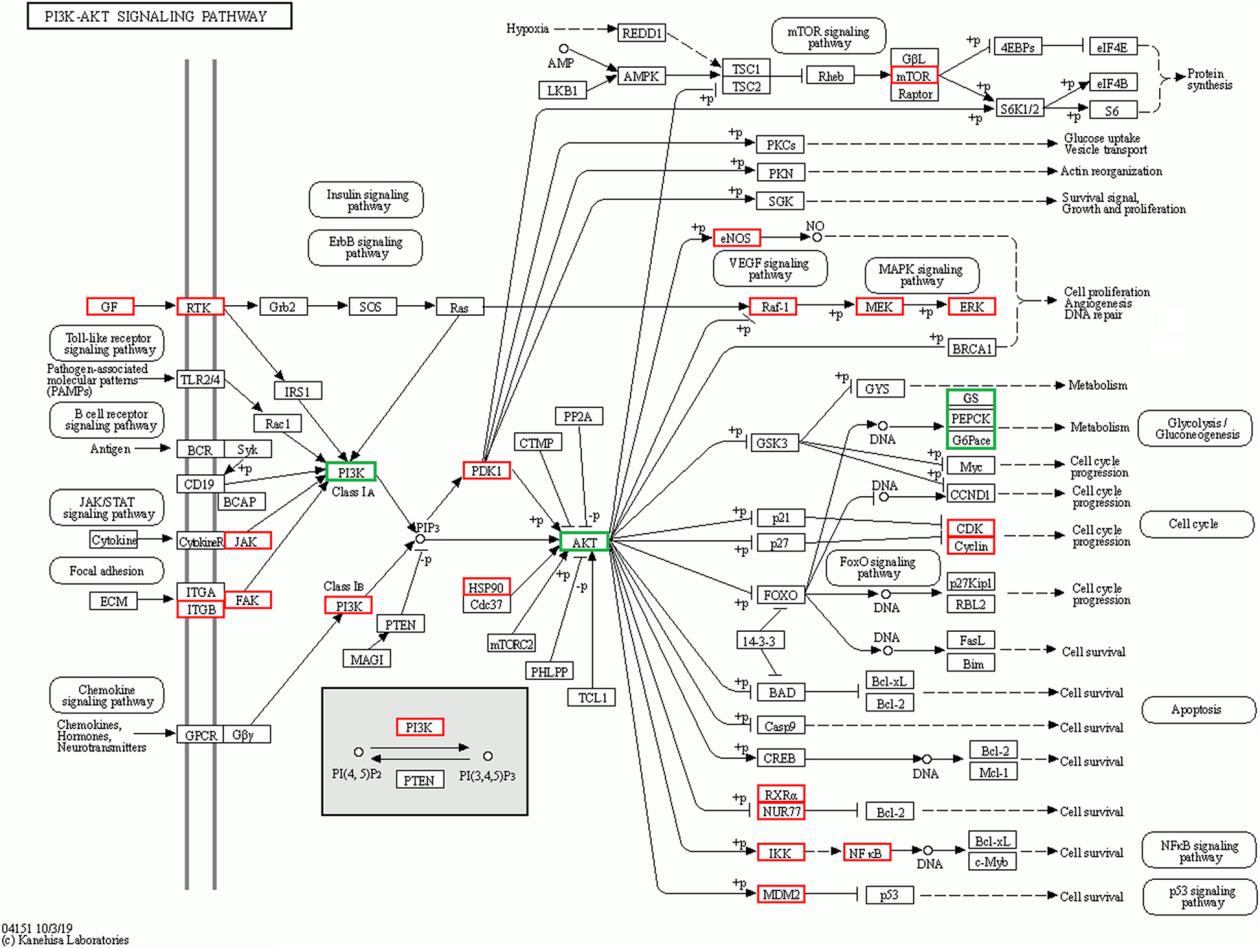
The potential mechanism of HYD against diabetes involved in the PI3K-AKT signaling pathway. The nodes that appear red represent potential target proteins of HYD, and the nodes that appear light represent relevant targets in the pathway, the nodes that appear green in represent core targets and glucose metabolism-related targets in the cell experiments

To gain further insight into the biological functions of the putative genes involved in the effect of HYD against diabetes, functional enrichment analyses were performed in our study. In the biological processes (BP) category, these target genes showed significant enrichment in the response to chemical, regulation of biological quality, response to organic substance, cellular response to chemical stimulus, etc. (Fig. 3A); in the cellular components (CC) category, these target genes showed significant enrichment in the vesicle, plasma membrane part, cell surface, cytosol, endomembrane system, etc. (Fig. 3B); in the molecular functions (MF) category, these target genes showed significant enrichment in the catalytic activity, anion binding, small molecule binding, drug binding, etc. (Fig. 3C). As shown in Fig. 4A, B, the top 10 items were identified based on the KEGG analysis with *p*-values < 0.05, mainly included MAPK signaling pathway, PI3K-AKT signaling pathway, Rap1 signaling pathway, progesterone-mediated oocyte maturation, central carbon metabolism in cancer, endocrine resistance, acute myeloid leukemia, EGFR tyrosine kinase inhibitor resistance, regulation of lipolysis in adipocytes, and VEGF signaling pathway. Combined with previous studies, we speculated that the PI3K-AKT signaling pathway may play a vital role in the treatment of diabetes by HYD (Fig. 5).

### Identification of core targets of HYD in treating diabetes

**Fig. 6:**
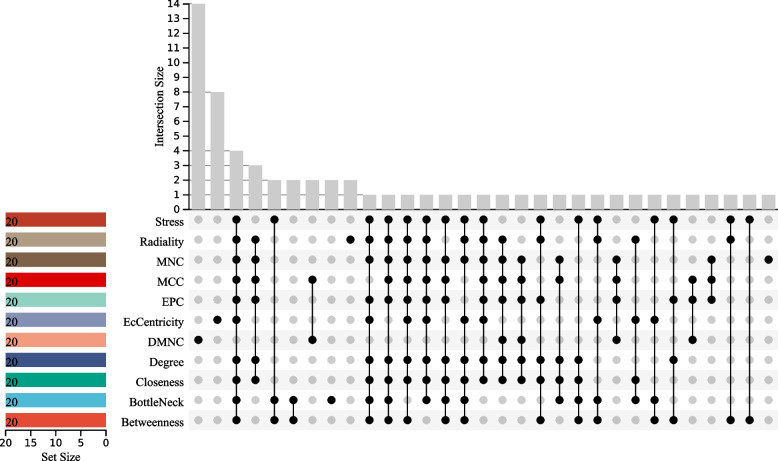
Identification of core targets of HYD in treating diabetes

Figure 6 presents the top 20 genes identified through screening by 11 topological analysis algorithms in cytoHubba. Using the R package “UpSet”, we were able to identify four core genes, namely HSP90AA1, AKT1, SRC, and MAPK1.

### Molecular docking

**Fig. 7:**
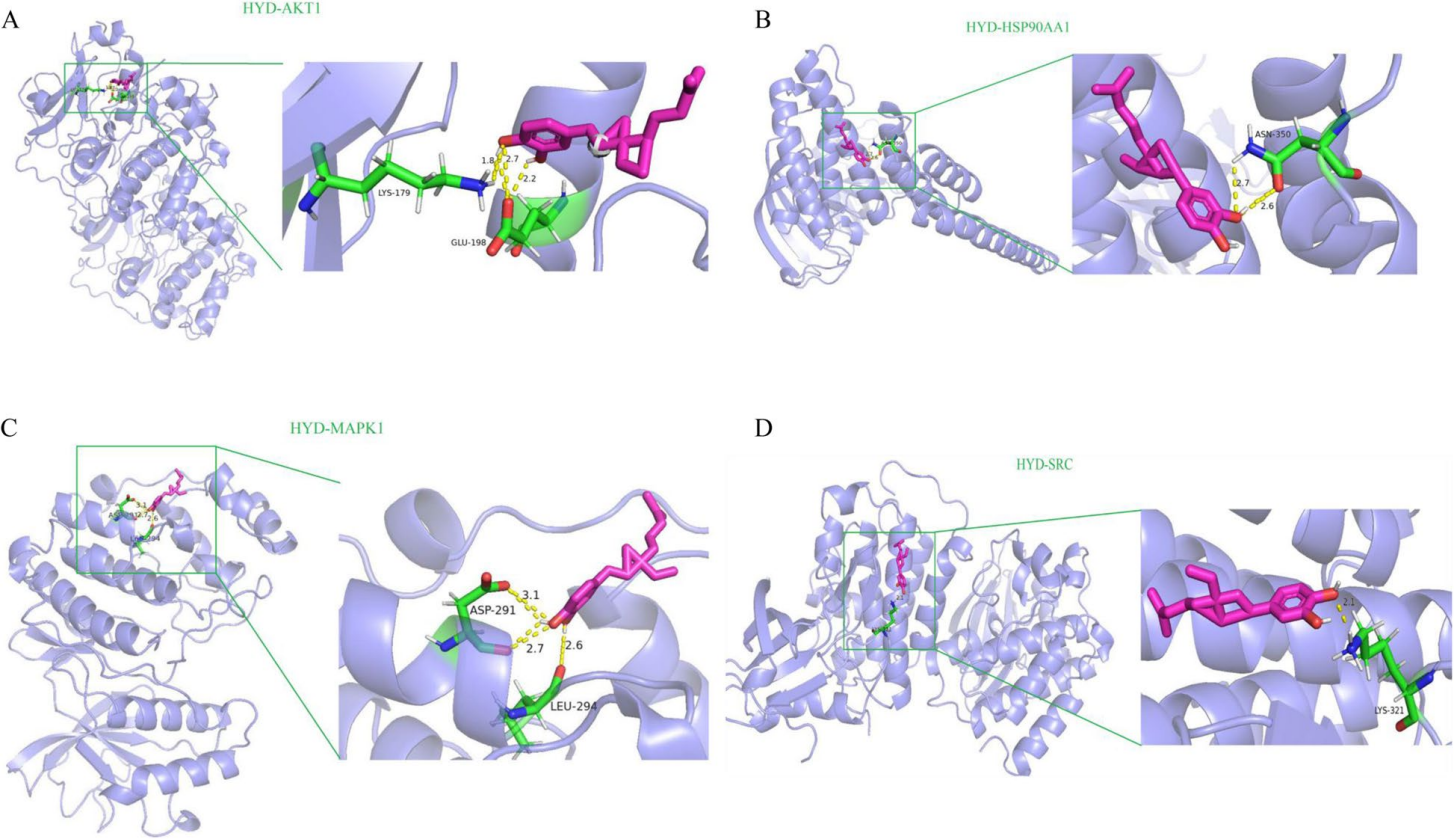
Molecular docking results of HYD with core targets. **A** HYD-AKT1; **B** HYD-HSP90AA1; **C** HYD-MAPK1; **D** HYD-SRC

Molecular docking studies were conducted to investigate the interactions between HYD and four potential target genes (HSP90AA1, AKT1, SRC, and MAPK1) at a molecular level. The AutoDock Tools software was employed for these studies, resulting in a total of four docking results. Among these results, one docking score below − 5.0 kcal/mol was obtained. The docking score, which is presented in Table S2, indicates the strength of binding between the compound and the core protein. It is noteworthy that AKT1 exhibited a particularly significant role in diabetes treatment, as it exhibited a strong binding affinity to HYD, with a binding energy below − 5.0 kcal/mol. In addition, the visualizations of the lowest binding energies between core targets and HYD were produced using PyMOL. HYD forms hydrogen bonds with the amino acid residues LYS-179 and GLU-198 when interacting with the AKT1 receptor protein (Fig. 7A); HYD forms hydrogen bonds with the amino acid residue ASN-350 when interacting with the HSP90AA1 receptor protein (Fig. 7B); HYD forms hydrogen bonds with the amino acid residues ASP-291 and LEU-294 when interacting with the MAPK1 receptor protein (Fig. 7C); HYD forms hydrogen bonds with the amino acid residue LYS-321 when interacting with the SRC receptor protein (Fig. 7D).

### Prediction of ADMET properties of HYD

In this study, we utilized pkCSM to evaluate the ADMET properties of HYD. The results presented in Table S3 revealed that HYD exhibited a human intestinal absorption rate of 91.16, indicating favorable absorption. To assess the distribution of HYD within the brain, we examined its blood-brain barrier (BBB) permeability, measured by the Log BB value. The Log BB result of 0.177 indicated that HYD could effectively penetrate the BBB. Furthermore, our findings suggest that HYD does not inhibit the activity of CYP2D6, CYP3A4, and CYP2C9 enzymes, implying that it is unlikely to interfere with the clearance and metabolism of drug substrates processed by these enzymes. Additionally, HYD demonstrated a renal total clearance rate of 0.312 and is not expected to serve as a substrate for organic cation transporter 2 (OCT2). Lastly, the toxicity data provided promising evidence of the safety profile of HYD.

### Effect of HYD on the viability of HepG2 cells

**Fig. 8:**
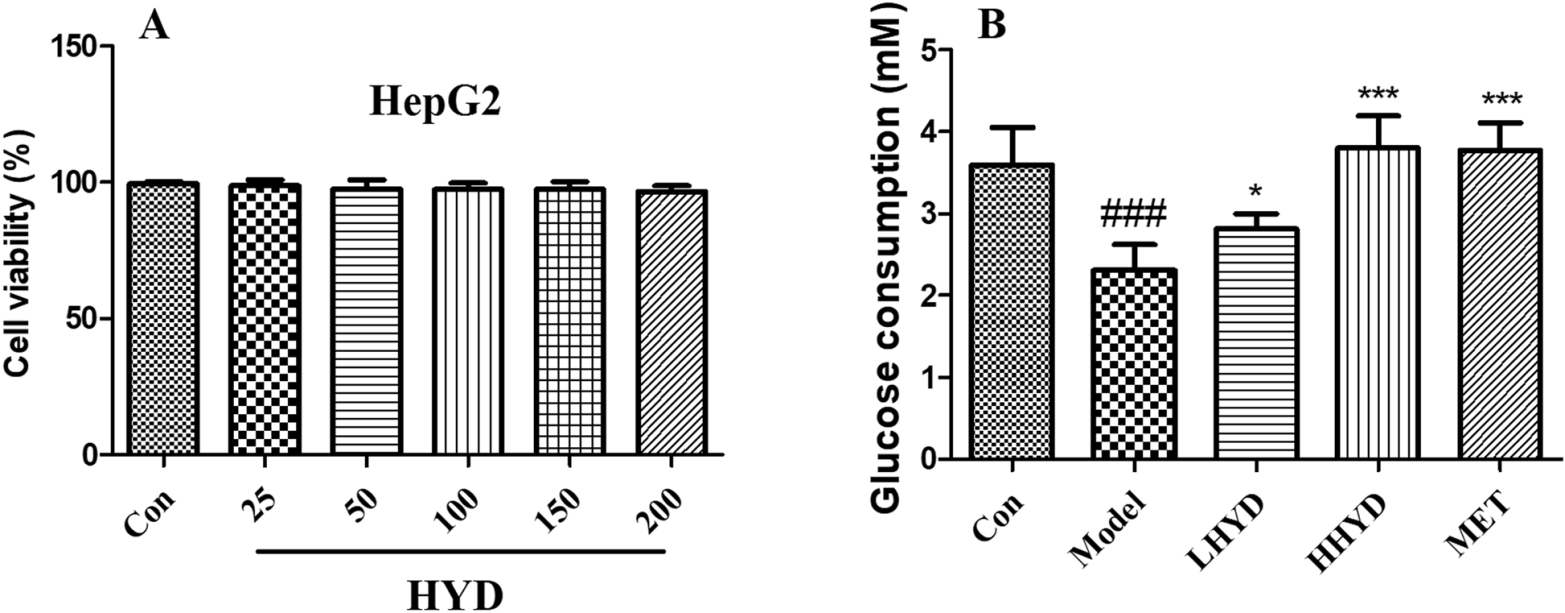
**A** Cell viability at different concentrations of HYD on HepG2 cells. **B **Effect of HYD on glucose consumption in insulin resistance model (HepG2 cells). HepG2 cells were exposed to 100 nM insulin and 25 mmol/L glucose only or with different concentrations of HYD for 24 h. The values were presented as the mean ± SD, with a sample size (n) of 6 for each group. Group comparisons were conducted using a one-way analysis of variance. ### denotes *P* < 0.001, vs. the control group; * denotes *P* < 0.05, *** denotes *P* < 0.001, vs. the model group. # denotes the comparison of the model group with the normal group; * denotes the comparison of the drug group with the model group

The CCK8 assays were used to assess the cytotoxic effects of HYD. As shown in Fig. 8A and Figure S1A, the viability of HepG2 cells and C2C12 cells remained unaffected when treated with HYD at concentrations ranging from 25 to 200 µg/mL.

### Effect of HYD on glucose consumption in insulin resistance model

As illustrated in Fig. 8B and Figure S1B, the model group exhibited significantly lower glucose consumption compared to the control group (*P* < 0.001). Both HYD and metformin were found to significantly elevate glucose consumption in insulin resistant HepG2 cells and C2C12 cells, with statistical significance observed at *P* < 0.05 or *P* < 0.001.

### Effect of HYD on the PI3K/Akt signaling pathway in insulin resistance model

**Fig. 9:**
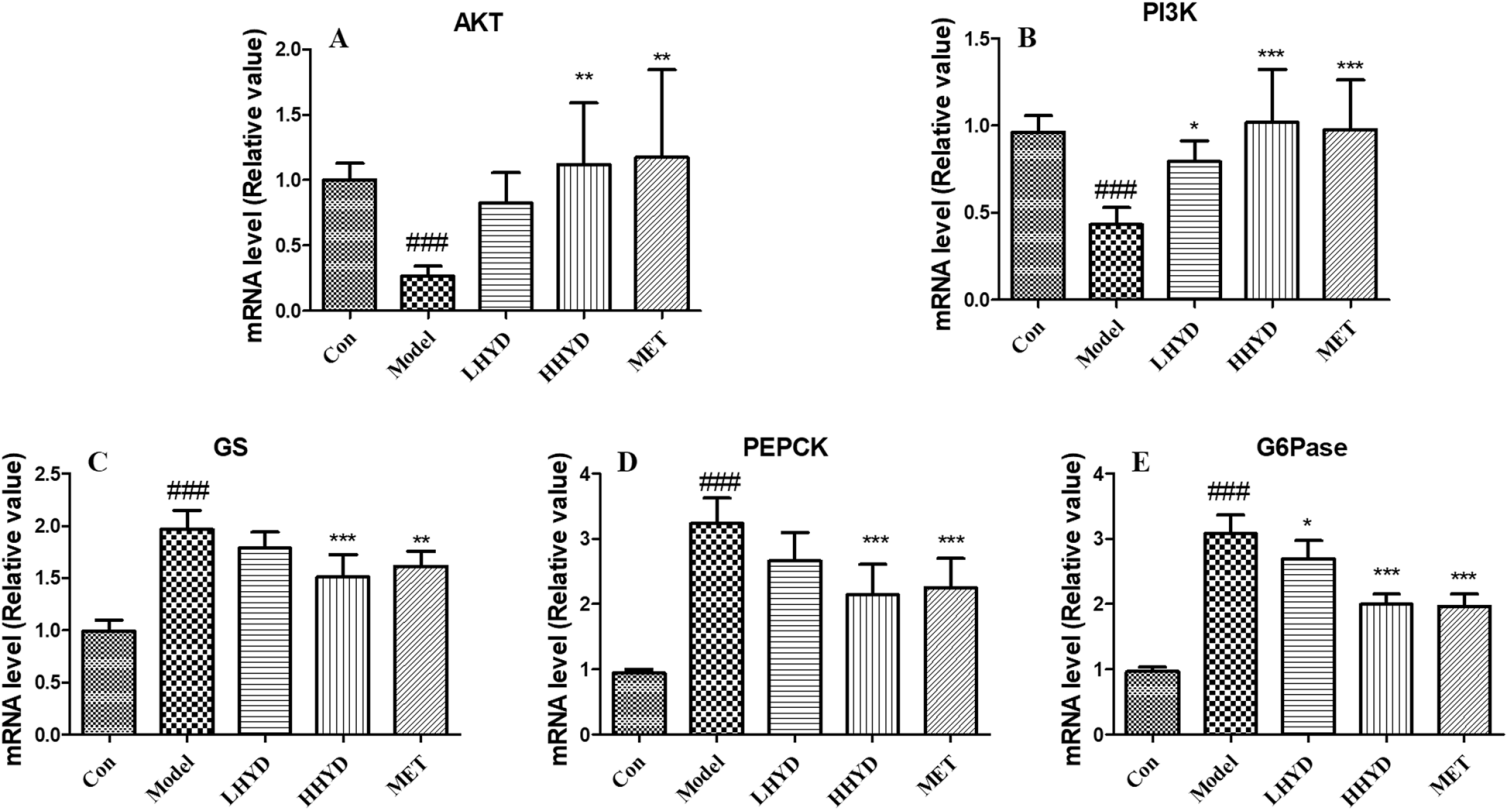
Effect of HYD on the PI3K/Akt signaling pathway in insulin resistance model (HepG2 cells). The relative mRNA level of AKT (**A**), PI3K (**B**), GS (**C**), PEPCK (**D**), and G6Pase (**E**) was measured by qRT-PCR analysis. HepG2 cells were exposed to 100 nM insulin and 25 mmol/L glucose only or with 25 and 50 µg/mL of HYD for 24 h. The values were presented as the mean ± SD, with a sample size (n) of 6 for each group. Group comparisons were conducted using a one-way analysis of variance. ### denotes *P* < 0.001, vs. the control group; * denotes *P* < 0.05, ** denotes *P* < 0.01, *** denotes *P* < 0.001, vs. the model group. # denotes the comparison of the model group with the normal group; * denotes the comparison of the drug group with the model group

In this study, we utilized qRT-PCR to examine the impact of HYD on the mRNA expression of AKT, PI3K, GS, PEPCK, and G6Pase (Fig. 9 and Figure S2). Compared to the model group, the HYD group exhibited increased mRNA levels of PI3K and Akt, while the mRNA levels of GS, PEPCK, and G6Pase were significantly reduced (*P* < 0.01 or *P* < 0.001).

## Discussion

Type 2 diabetes mellitus is a chronic endocrine and metabolic disorder associated with severe complications. The development of type 2 diabetes mellitus is multifactorial, involving both environmental and genetic factors, along with insulin resistance, which poses significant clinical challenges. Recently, there has been increasing interest in exploring new therapeutic strategies for type 2 diabetes mellitus, including the use of traditional medicine as a complementary treatment [27–29]. Previous studies showed that HYD exerted anti-alpha-glucosidase activity in vitro [18]. Nevertheless, the precise mechanism by which HYD exerts its therapeutic effects in diabetes remains unclear. Therefore, in this study, we employed a combination of network pharmacology analysis, molecular docking, and cell experiments to elucidate the potential targets and underlying mechanisms of action of HYD in treating diabetes.

In this study, a comprehensive approach was employed to predict the interactions between HYD and its potential protein targets. Targets from publicly available databases on diabetes were integrated, and the various signaling pathways and networks in which HYD targets are involved were explored. Docking studies were also conducted to predict specific interactions between HYD and its predicted protein targets. The results of molecular docking demonstrated that hydrogen bonding was the primary mode of interaction for HYD. In addition, the cytoHubba plug-in has identified four key targets (HSP90AA1, AKT1, SRC, and MAPK1) that have a significant connection to the pathogenesis and treatment of type 2 diabetes mellitus. HSP90AA1 was previously identified as a node in a protein-protein interaction network in diabetes through bioinformatics analysis [30, 31]. There is compelling evidence indicating that there are abnormalities in Akt signaling during the development of insulin resistance, which is a key aspect of the pathophysiology of Type 2 Diabetes Mellitus [32]. AKT1, the main isoform of AKT, is widely expressed in different tissues. One of the important targets of AKT is glycogen synthase kinase 3 (GSK3), which is essential in insulin signaling. In the insulin signaling pathway, GSK3 is inhibited by PI3K/AKT-mediated phosphorylation, resulting in increased glycogen synthesis and glucose disposal [33]. SRC plays a critical role in glucose-induced insulin secretion in pancreatic β-cells by regulating the subcellular localization and activity of glucokinase [34]. In microvascular endothelial cells from type 2 diabetic subjects, there was an increase in phosphorylation of the MAPKs ERK1/2 in the basal state [35]. Furthermore, HYD possesses a comparable configuration to bakuchiol. Prior research has demonstrated that bakuchiol exerted diverse pharmacological effects via the PI3K/Akt signaling pathway [36–38]. These investigations further substantiated that the antidiabetic efficacy of HYD is accomplished through the PI3K/Akt pathway.

Based on the results of KEGG pathway, HYD may treat diabetes through multiple pathways, such as MAPK signaling pathway, PI3K-AKT signaling pathway, endocrine resistance, EGFR tyrosine kinase inhibitor resistance, regulation of lipolysis in adipocytes, VEGF signaling pathway, etc. The PI3K/AKT signaling pathway is crucial in cellular physiology as it helps transmit growth factor signals during organismal growth and essential cellular processes. These processes encompass cell proliferation, cell survival, and protein synthesis, as well as glucose homeostasis and lipid metabolism [39]. Impaired PI3K/AKT-mediated glucose transport and glycogen synthesis are significant contributors to the pathogenesis of type 2 diabetes mellitus and obesity [40]. The pancreas consists primarily of four types of cells, with pancreatic β cells, playing a crucial role in the regulation of glucose homeostasis [41]. Activation of the PI3K/AKT pathway enhances insulin secretion from pancreatic β cells [42]. In this study, we conducted an in vitro qRT-PCR analysis to confirm the impact of HYD on the PI3K/Akt signaling pathway in HepG2 cells. The results indicated that HYD activates the PI3K/Akt signaling pathway, leading to enhanced glycogen metabolism and insulin resistance, ultimately resulting in a decrease in blood glucose levels. While our study did validate the potential anti-diabetic activity of HYD, these findings must be corroborated through animal experimentation.

## Conclusion

In conclusion, this study utilized cell experiments, network pharmacology, and molecular docking to investigate the potential mechanism of HYD in treating diabetes. The findings offered a preliminary understanding of how HYD may work and provided a new avenue for further research on its mechanism in diabetes treatment. Additionally, these results serve as a theoretical foundation for the clinical application of HYD in the treatment of diabetes.

### Supplementary Information

**Additional file 1: Table S1. **Sequences of primers used quantitative real-time PCR. **Table S2.** Binding energy between HYD and four core targets. **Table S3.** ADMET parameters of HYD predicted using pkCSM. **Figure S1.** (A) Cell viability at different concentrations of HYD on C2C12 cells. (B) Effect of HYD on glucose consumption in insulin resistance model (C2C12 cells). ###*P* < 0.001, vs. the control group; **P< 0.01, ***P < 0.001, vs. the model group. **Figure S2.** Effect of HYD on the PI3K/Akt signaling pathway in insulin resistance model (C2C12 cells). The relative mRNA level of AKT (A), PI3K (B), GS (C), PEPCK (D), G6Pase (E) was measured by qRT-PCR analysis. ###*P*< 0.001, vs. the control group; *P < 0.05, **P < 0.01, ***P < 0.001, vs. the model group.

## Data Availability

All data are available from the corresponding author upon request. The targets of HYD have been obtained by using the following three databases: Super-PRED (https://prediction.charite.de/index.php), PharmMapper (http://www.lilab-ecust.cn/pharmmapper/), and SwissTargetPrediction (http://www.swisstargetprediction.ch/). The GeneCards (https://www.genecards.org/), Therapeutic Target Database (https://db.idrblab.net/ttd/), and Comparative Toxicogenomics Database (https://ctdbase.org/) were used to identify diabetes-related targets.
