# Supplementary material for: Integrated investigation and discovery of therapeutic targets for 3-hydroxybakuchiol against diabetes based on molecular docking studies and cell experiments
Source: BMC Complement Med Ther. 2023 Nov 29;23:431. doi: 10.1186/s12906-023-04248-6 (PMC10688491; doi:10.1186/s12906-023-04248-6)
Supplement: Supplementary file 1 — Additional file 1: Table S1. Sequences of primers used quantitative real-time PCR. Table S2. Binding energy between HYD and four core targets. Table S3. ADMET parameters of HYD predicted using pkCSM. Figure S1. (A) Cell viability at different concentrations of HYD on C2C12 cells. (B) Effect of HYD on glucose consumption in insulin resistance model (C2C12 cells). ###P < 0.001, vs. the control group; **P< 0.01, ***P < 0.001, vs. the model group. Figure S2. Effect of HYD on the PI3K/Akt signaling pathway in insulin resistance model (C2C12 cells). The relative mRNA level of AKT (A), PI3K (B), GS (C), PEPCK (D), G6Pase (E) was measured by qRT-PCR analysis. ###P< 0.001, vs. the control group; *P < 0.05, **P < 0.01, ***P < 0.001, vs. the model group. [file 12906_2023_4248_MOESM1_ESM.docx]

Table S1 Sequences of primers used quantitative real-time PCR.

| Gene | Forward primer (5' to 3') | Reverse primer (5' to 3') |
| --- | --- | --- |
| AKT | GCCTCTGCTTTGTCATGGAG | AGCATGAGGTTCTCCAGCTT |
| PI3K | CCAGACCAGTACGTTCGAGA | GAAACTGCCCTATCCTCCGA |
| GS | CAACCACTAGGAGGGAGATCC | TCATCTTGGTGTGGGTGTAAAAG |
| PEPCK | CTTTGGAGGCCGTAGACCTG | GCCTTTATGTTCTGCAGCCG |
| G6Pase | CATTGACACCACACCCTTTGC | CCCTGTACATGCTGGAGTTGAG |
| β-actin | GGACTTCGAGCAAGAGATGG | AGCACTGTGTTGGCGTACAG |

Table S2 Binding energy between HYD and four core targets.

| Drug | Binding Energy (kcal/mol) | | | |
| --- | --- | --- | --- | --- |
|  | HSP90AA1 | AKT1 | SRC | MAPK1 |
| HYD | -3.35 | -6.56 | -3.16 | -4.6 |

Table S3 ADMET parameters of HYD predicted using pkCSM.

| Property | Model Name | Predicted Value | Unit |
| --- | --- | --- | --- |
| Absorption | Intestinal absorption (human) | 91.16 | Numeric (% Absorbed) |
| Distribution | BBB permeability | 0.177 | Numeric (log BB) |
| Metabolism | CYP2D6 inhibitor | No | Categorical (Yes/No) |
|  | CYP2C9 inhibitor | No | Categorical (Yes/No) |
|  | CYP3A4 inhibitor | No | Categorical (Yes/No) |
| Excretion | Total Clearance | 0.312 | Numeric (log ml/min/kg) |
|  | Renal OCT2 substrate | No | Categorical (Yes/No) |
| Toxicity | Oral Rat Acute Toxicity (LD50) | 2.439 | Numeric (mol/kg) |
|  | Oral Rat Chronic Toxicity (LOAEL) | 2.326 | Numeric (log mg/kg bw/day) |
|  | AMES toxicity | No | Categorical (Yes/No) |
|  | Hepatotoxicity | No | Categorical (Yes/No) |
|  | Max. tolerated dose (human) | 0.427 | Numeric (log mg/kg/day) |


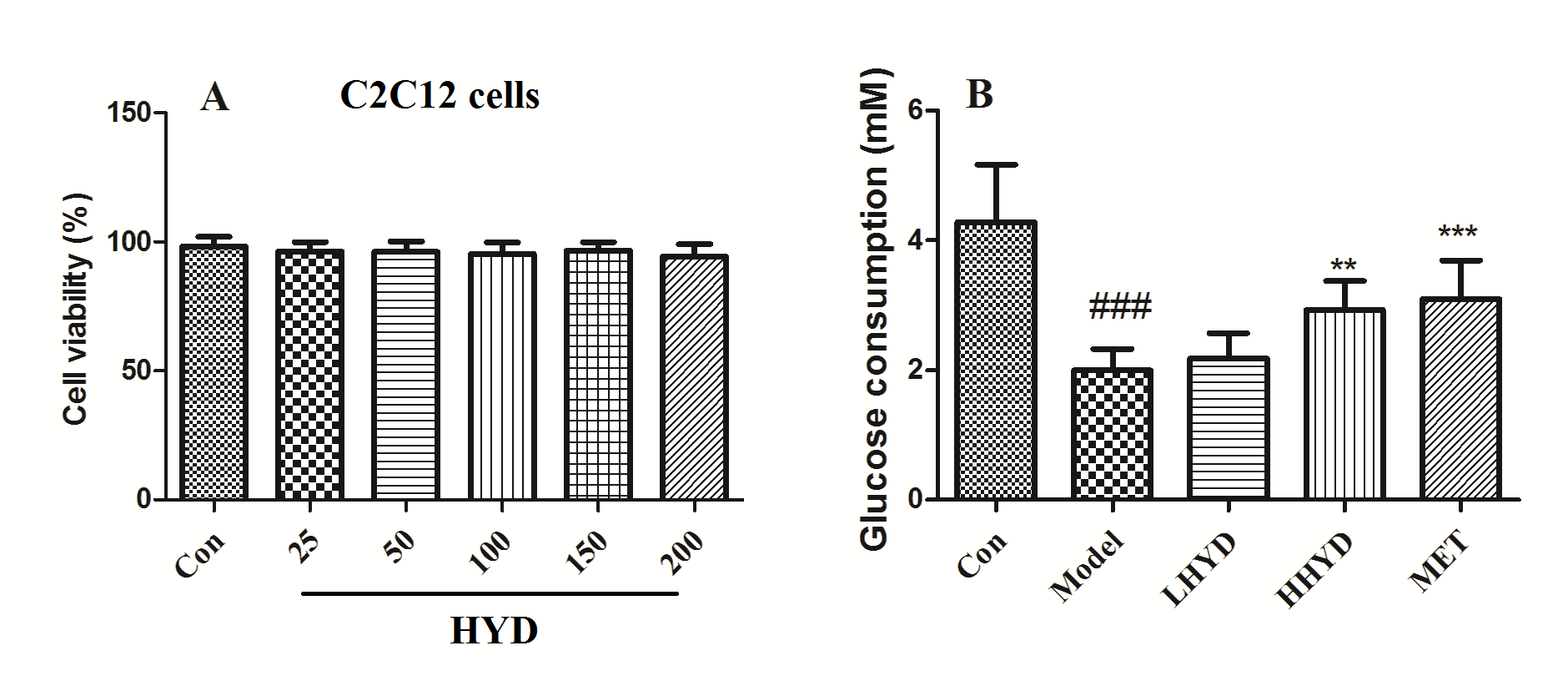


Figure S1 (A) Cell viability at different concentrations of HYD on C2C12 cells. (B) Effect of HYD on glucose consumption in insulin resistance model (C2C12 cells). ###P < 0.001, vs. the control group; **P < 0.01, ***P < 0.001, vs. the model group.


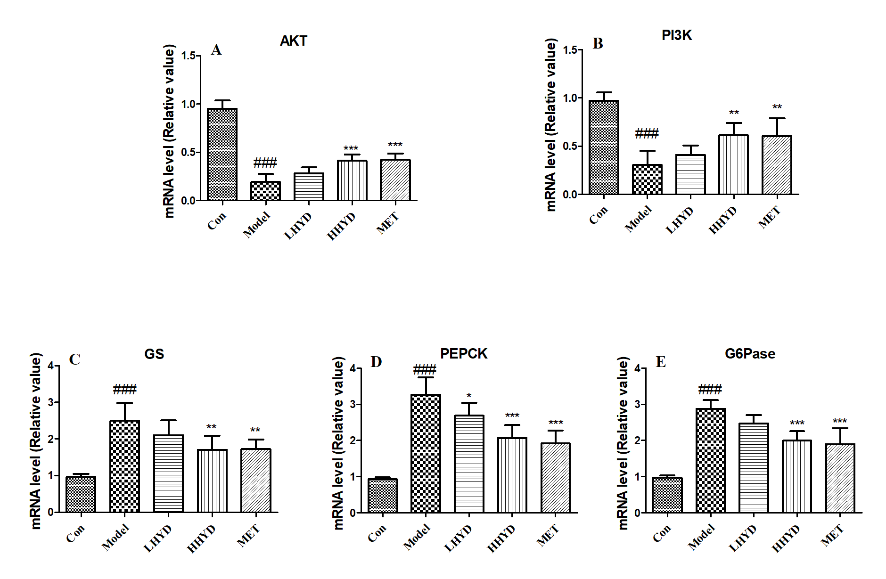


Figure S2 Effect of HYD on the PI3K/Akt signaling pathway in insulin resistance model (C2C12 cells). The relative mRNA level of AKT (A), PI3K (B), GS (C), PEPCK (D), G6Pase (E) was measured by qRT-PCR analysis. ###P < 0.001, vs. the control group; *P < 0.05, **P < 0.01, ***P < 0.001, vs. the model group.
